# Early Operation for Hare-lip

**Published:** 1875-06

**Authors:** E. R. Maxson

**Affiliations:** Syracuse, New York


### EARLY OPERATION FOR HARE-LIP.

BY E. R. MAXSON, M. D., OF SYRACUSE, NEW YORK.

In August of 1874, at the birth of a child with a fissure of the upper lip, on the left side, extending nearly to the nose, and involving the superior maxillary and palate bones, I was requested, by very anxious parents, to operate as soon as possible for the relief of the deformity which was really great, and to them intolerably shocking.

I selected the eighth day after birth, taking everything connected with the case, including the chances for the child, and the feelings of the parents into the account, and without chloroform, being assisted only by the nurse and another lady (the grandmother.) I proceeded to operate in the usual way, by detaching the upper lip from the bones behind, paring the edges of the fissure from above downwards, carrying the knife inwards, saving the bottom of the cleft to prevent a notch; detaching the upper angle with sharp scissors, passing three small silver needles through about two thirds of the thickness of the lips, commencing at the lower angles to make the adaptation exact, applying the twisted suture, commencing with the uper needle and cementing the suture and lip by collodion, as suggested by Fergusson, applying long strips of adhesive plaster made narrow in the middle to fit between the needles, and extending back across the cheeks; and finally, by placing a compress on each cheek to aid in keeping the two parts of the cleft lip together, and to aid in approximating the divided palate and superior maxillary bones; kept in place by a roller moderately tight, passed around the head, being stitched together to prevent slipping.

The child appeared to suffer but little during the operation, and indeed, afterwards. The dresssings kept in place, and except for the cutting of the upper adhesive plaster by the nurse, during my absence, to make the child " nurse and breathe better," as she said, no accident occurred. I removed the pins on the fourth day, and all the dressings on the fourteenth day, only a small point at the upper part having filled up by granulations, the remainder having united by the adhesion before the removal of the pins and reapplying of the adhesive plasters on the fourth day.

The child appeared tolerably well, but did not grow much for several weeks. But later it commenced to grow, and now, being about six months old, it is represented as being very large and the picture of health, and presenting little or no deformity, or even marks of the operation. In fact, I believe that no one unacquainted with the *facts* has ever suspected that there had been a hare-lip, or an operation of any kind upon the child.—*Medical Record.*

---

*Hydrate of Chloral in Hyper-Emesis*—Dr. D. B. Simmons, of Yokohama, Japan, and Dr. H. Morse, of New York city, laud the efficiency of this drug as an anti-emetic in the puerperal condition, administered *per enema.* Instances are cited in which very satisfactory results were obtained by the use of twenty to sixty grains, repeated if required, after the ineffectual employment of various other remedies.—*N. Y. Med. Record.* J. B. M.

